# Cell-Sonar, a Novel Method for Intracellular Tracking of Secretory Pathways

**DOI:** 10.3390/cells13171449

**Published:** 2024-08-29

**Authors:** Sabrina Brockmöller, Thomas Seeger, Franz Worek, Simone Rothmiller

**Affiliations:** Bundeswehr Institute of Pharmacology and Toxicology, 80937 Munich, Bavaria, Germany

**Keywords:** cellular tracking, nicotinic acetylcholine receptor, protein homeostasis

## Abstract

Background: Intracellular tracking is commonly used in trafficking research. Until today, the respective techniques have remained complex, and complicated, mostly transgenic target protein changes are necessary, often requiring expensive equipment and expert knowledge. Methods: We present a novel method, which we term “cell-sonar”, that enables the user to track expression changes of specific protein markers that serve as points of interaction. Our study provides comparable analyses of expression changes of these marker proteins by in-cell Western analyses in two otherwise isogenic cell lines that only differ in the overexpression of the tracked target protein. Using the overexpressed human adult muscle-type nicotinic acetylcholine receptor as an example, we demonstrate that cell-sonar can cover multiple intracellular compartments such as the endoplasmic reticulum, the pathway between it and the Golgi apparatus, and the endocytic pathway. Results: We provide evidence for receptor maturation in the Golgi and storage in recycling endosomes, rather than the fate of increased insertion into the plasma membrane. Additionally, we demonstrate with the implementation of nicotine that the receptor’s destiny is exasperated up to secondary degradation. Conclusions: Cell-sonar is an affordable, easy-to-implement, and cheap method that can be adapted to a broad variety of proteins and cellular pathways of interest to researchers.

## 1. Introduction

The intracellular tracking of secretory pathways is of broad interest in cell biology for researchers. Investigations of these pathways are often complicated, elaborate, and require sophisticated technologies, which are expensive. Among these techniques are confocal laser scanning microscopy [1], Förster resonance energy transfer [2], total internal reflection fluorescence microscopy [3], and fluorescence-activated cell sorting [4]. The target proteins are typically assessed for only one aspect. Further, genetic modifications like fluorescent tags are often required, thereby further enhancing the effort required. Overviews of pathway networks are usually possible by use of proteomics, metabolomics, or genomic screens [5]. These methods are specialized and suitable for their applications, but a simple, quick, and cheap method is still needed. Western blot [6], glycosylation digestion [7], or in-cell Western (ICW) [8] are methods that have those properties, but it is difficult to get a quick, broad overview of these methods. A novel method that combines specific detection, fast feasibility, easy application, and low costs is lacking. This study proposes a new approach to intracellular tracking while observing one protein through the secretory pathway from the endoplasmic reticulum (ER) to the plasma membrane and back. We called the method cell-sonar because it does not track the protein itself, but rather individual cellular interaction points by specific marker proteins. These marker interaction points together provide a first overview of whether the protein remains or passes.

The proposed cell-sonar is based on knowledge of subcellular pathway occurrences. Proteins are synthesized in the ER and associated with chaperones that support folding, misfold degradation, and ATP supplementation for energy-dependent procedures [9]. Proteins exit the ER to their specific destinations in an ordered manner by ER entry site-organized proteins [10,11]. ER translocation is a controlled process performed by COPII vesicles [12] at ER entry sites [13], which consist of Sec24 isoforms [14] that are required for specific interaction with the following compartments, such as the ER–Golgi-intermediate compartment (ERGIC) and the Golgi [15]. ERGIC grows through a homotopic fusion of COPII vesicles [16,17]. Vesicle budding and fusion processes from compartment to compartment are cargo-dependent [18] and have been orchestrated by the GTPase Rac1 [19,20]. The processes of retention in the retrograde transfer of cargo proteins occur via a similar vesicle trafficking model as COPI, which consists of coat proteins [21,22]. The cargo proteins need to associate with numerous interaction proteins to leave the ER and enter the Golgi apparatus.

After Golgi, the trafficking is targeted to specific destinations, which could include plasma membrane insertion. The endocytic pathway, after reaching the destination of the plasma membrane, is also strictly regulated. Early endosomes are a possible starting point for a controlled sorting process employed to deliver cargo proteins [23] to the trans-Golgi network [24,25], late endosomes [26,27] with subsequent secondary degradation, or recycling endosomes [28,29] for cargo storage. Modifications of endosomal Rab GTPases determine the fate of cargo proteins. The secretory pathway in mammalian cells is a cross-linked network of complex, ordered succession events. Several links, interactions, or regulatory points are still unknown today. In our study, we want to use our novel method, cell-sonar, to elucidate these pathways for initial protein localization using detectable protein–protein interactions.

To demonstrate how to apply the cell-sonar technique in resolving a particular unknown cellular pathway, we focus our study on the human adult muscle-type α1_2_β1δε nicotinic acetylcholine receptor (nAChR), which serves as an example for our study. In recent decades, numerous research groups have extensively examined the nAChR, and the authors have reported that the surface expression in the plasma membrane was reduced compared to intracellular expression in different contexts [30,31,32,33]. We previously reported the generation of a new transgene cellular model system for nAChR, and confirmed in our work that nicotine treatment increased the number of nAChR subunits intracellularly, but this effect was not detected on the cell surface [34]. Until now, the inefficient biogenesis of the receptor was attributed to its degradation, but without further evidence for this hypothesis. The new approach of intracellular tracking with cell-sonar should thus clarify what happens to the receptor within the cell. We hypothesize that our proposed novel method, cell-sonar, will provide a first insight into the subcellular localization of the nAChR.

## 2. Materials and Methods

### 2.1. Cell Culture

Previously, we reported on a Chinese hamster ovary (CHO) cell line that stably expressed recombinant α1_2_β1δε nAChR [34]. These cells were incubated in F-12 Nut Mix + GlutaMaxTM (31765-027 Gibco, Karlsruhe, Germany) with 16.5% fetal calf serum (FCS) (10270-106 Gibco, Karlsruhe, Germany) at 37 °C, 5% CO_2_, and 90% humidity. At a confluence of 50–70%, the cells were passaged with PBS 7.4 pH (10010-015 Gibco, Karlsruhe, Germany) used for washing and TrypLETM Express (12605-010 Gibco, Karlsruhe, Germany) for detaching. The untransduced CHO cells showed the same growth conditions, except that FCS supplementation was 9% instead of 16.5%.

### 2.2. Cell-Sonar for Intracellular Tracking with ICW

ICW was performed under four different conditions: (i) untransduced CHO cells compared to (ii) transduced CHO cells with nAChR, and (iii) untransduced CHO cells treated with 30 µM nicotine for 24 h in medium compared to (iv) transduced CHO cells with nAChR treated with 30 µM nicotine for 24 h in medium. ICW was performed with 30,000 cells per well seeded in black 96-well microplates with clear bottoms. The cells were fixed in wells after 24 h and permeabilized for 10 min with ice-cold 100% methanol at room temperature. Afterwards, the methanol was removed, and the plates were washed three times with 150 µL/well PBS for 5 min. The cells were blocked for 1.5 h with a blocking buffer (LiCor, Bad Homburg, Germany) at 180 rpm at room temperature. Then, the blocking buffer was removed, and the cells were incubated for 2.5 h with the primary antibody diluted 1:200 in blocking buffer at 180 rpm at room temperature. The following primary antibodies were used: CN ab133615, BiP ab213258, Sil1 ab228868, Hrd1 ab249578, UGGT1 ab124879, COPII Sec24A ab262869, GAPDH ab8245, COPII Sec24B ab122445, COPII Sec24C ab122633, COPII Sec24D ab122732, COPIα ab181224, COPIβ ab2899, ERGIC53 ab125006, Rab5 ab231095, Rab7 ab137029, Rab4 ab226047, Rab11 ab232655, calmodulin ab214793, vps26 ab181352 and vps35 ab240141 (all Abcam, Cambridge, UK). The plates were washed three times for 5 min with PBST (0.1% Tween-20 in PBS) after incubation. The secondary antibody was incubated for 1 h, diluted at 1:800 in blocking buffer at 180 rpm and protected from light. As a secondary antibody IRDye 800 Rabbit 92632211, and for nuclear and cytoplasm staining Cell Tag Stain 700 926-41090 (both LiCor, Bad Homburg, Germany), were used to normalize emission signals to cell counts. At last, the cells were washed three times for 5 min with PBST. Every well was filled with 100 µL PBS and Odyssey CLx (LiCor, Bad Homburg, Germany) was used for scanning ICW plates at 800 and 680 nm. Images were analyzed with Image Studio software version 5.2 (LiCor, Bad Homburg, Germany) and fluorescence signals were normalized to cell counts.

### 2.3. Glycosylation Digest and Western Blot

The shift in the glycosylation digest of the δ and ε subunits of the nAChR was detected with SDS PAGE and Western blot. Therefore, CHO cells were solubilized and purified as previously reported [34]. Digestion was performed with PNGase F and Endo H (P0704L, P0702L both by New England Biolabs, Frankfurt, Germany). For each enzyme, at least 1620 ng of the sample was incubated with 3 µL denaturation buffer for 10 min at 100 °C. Afterwards, the sample was cooled on ice, and 2500 units of Endo H and 3 µL buffer 3 or 2500 units PNGase F with 3 µL buffer 2 and 3 µL 1% NP-40 solution were incubated for 3 h at 37 °C.

Per SDS gel well, 100–200 ng digested nAChR was mixed with loading buffer (2% SDS, 0.5 M DTT, 5 µL 4X loading dye by LiCor), incubated for 10 min at 60 °C, and applied. SDS PAGE and Western blot were performed as previously reported [34]. The subunits were detected with antibodies δ ab233758 and ε ab233831 (both Abcam, Cambridge, UK) diluted 1:1000. Membranes were scanned with Odyssey CLx, and the images were analyzed with Image Studio software version 5.2 (both LiCor, Bad Homburg, Germany).

### 2.4. Endosome Isolation and Enrichment of Rab11 Marked Endosomes

For endosome isolation, the Endosome Isolation and Cell Fractionation Kit (IBT-ED-028 by Biozol, Eching, Germany) was used. At least 4 × 10^6^ CHO cells, either transduced with nAChR or untransduced, were harvested separately and lyzed according to the manufacturer’s protocol. Endosome solutions were incubated in a ratio of 4:1 with buffer B at 4 °C overnight. Afterwards, endosomes were pelleted by centrifugation at 10,000× *g* for 1 h at 4 °C, resuspended in 20 µL PBS and stored at 4 °C.

To enrich Rab11-marked endosomes, Dynabeads Protein G (10004D Invitrogen, Darmstadt, Germany) was applied. Thus, 100 µL Rab11 ab232655 antibody (Abcam, Cambridge, UK) with a concentration of 1 mg/mL was added into 2 mL PBST (0.05% Tween-20 in PBS) solution per 500 µL magnetic beads. The labeling of beads was performed for 15 min at 21 °C and 800 rpm in a heating block. The supernatant was removed with DynaMag2 (Invitrogen, Darmstadt, Germany) and the beads were carefully washed with 2 mL PBST. Labeled magnetic beads were stored at 4 °C in 500 µL PBST. For immunoprecipitation, 20 µL endosome samples were mixed with 50 µL antibody-labeled magnetic beads and filled up to 100 µL with PBS. Incubation was performed for 2 h at 21 °C and 800 rpm in a heating block. Afterwards, beads were washed three times with 200 µL PBS. Discarding of the supernatant was always performed with DynaMag2. Finally, endosomes were eluted from the beads with 20 µL 0.1 M Glycine pH 2.0 for 2 min, at 16 °C and 800 rpm, in a heating block. To restore a pH of 7.5, 2.25 µL 1 M Tris buffer pH 8.5 was added. Rab11 endosome solution was stored at 4 °C for one week.

### 2.5. Flow Cytometric Analysis of Rab11 Marked Endosomes

Primary antibodies His-tag ab18184 for α1 subunit (His-tagged) and β1 ab236959 (both Abcam, Cambridge, UK) were labeled using Alexa Fluor dyes 568 and 680 (A20184, A20188 by Invitrogen, Darmstadt, Germany), respectively. Therefore, both primary antibodies, each 100 µL with a concentration of 1 mg/mL, were separately desalted with Zeba Spin Desalting columns (89878 Invitrogen, Darmstadt, Germany) and labeled with the respective Alexa Fluor dyes according to manufacturers’ protocol. After labeling and purification, the dye-labeled primary antibodies were checked for their concentration, and labeling success was determined by the degree of labeling. They were stored in PBS with 1 mg/mL BSA at 4 °C and protected from light.

For FACS analysis, each sample of the isolated Rab11 endosomes was filled up to 500 µL with PBS. All samples were incubated with both labeled primary antibodies (diluted to 1:200 for His-Tag and 1:100 for β1) for 45 min at room temperature, and were protected from light. Afterwards, they were centrifuged at 1500× *g* for 5 min and the supernatant was discarded. Then, endosomes were washed with 500 µL PBS + 2% FCS by centrifugation. The endosomes were resuspended in at least 30 µL PBS + 2% FCS. All samples were analyzed using an ImageStreamX MarkII instrument (Luminex Corporation, Austin, TX, USA) using the 60× objective and recording 30,000 events. Single-stained samples were used for compensation. The software used for data acquisition was ISX (201.1.0.765), and for data analysis it was IDEAS (6.2.187.0) both AMNIS part of EMD Millipore (Seattle, WA, USA).

### 2.6. Statistics

Data analysis was performed using the exported data of Odyssey CLx on ICW results. GraphPad Prism 9.5.1 (733) software was used for graphic presentation and a two-sample *t*-test with dependent samples was applied. *p*-values were calculated and values below 0.05 were considered statistically significant.

## 3. Results

### 3.1. Concept of the Cell-Sonar Method

**Figure 1:**
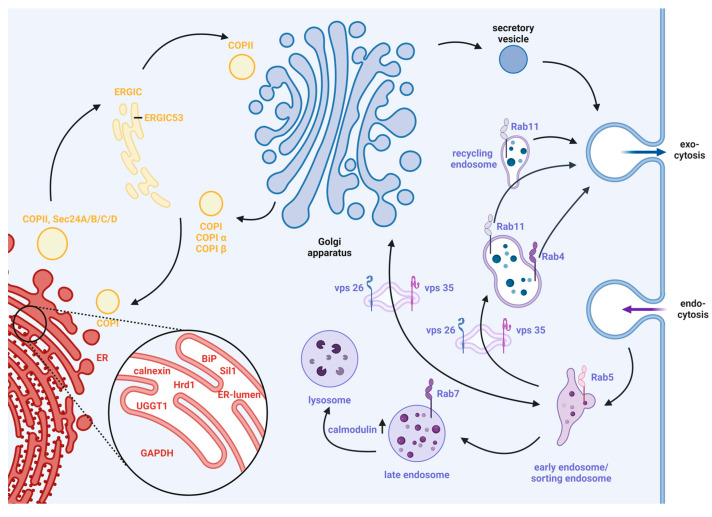
Illustration of the concept of the cell-sonar method and what protein interaction points are included. A summary of today´s known models of intracellular trafficking pathways is outlined in the text in Section 1 and Section 2.1 as a description of model details. The three selected areas are shown as a base of the concept of the novel cell-sonar method, and these were chosen according to the question of the nAChR fate. Within the endoplasmic reticulum (ER, red area), molecular chaperones (UGGT1, calnexin, Hrd1, BiP, Sil1) are needed, and GAPDH is needed outside of the ER for correct protein folding. The six interaction proteins give a first overview of the destination regarding calnexin cycle or ATP-dependent interactions. The secretory pathway between the ER and the Golgi apparatus (yellow area) provides an overview of where the secretion is directed (Sec24A/B/C/D) for anterograde trafficking associations, and COPIα/β and the ER–Golgi-intermediate compartment (ERGIC) (53) are needed for retrograde trafficking associations. The post-Golgi endocytic pathway (purple area) consists of destinations between secondary degradation (Rab5/7 and calmodulin) and storage (Rab4/11, vacuolar protein sorting (vps) 26/35). Created with BioRender.com (accessed on 1 May 2024).

Our study illustrates how the method works. We divided the whole cell into areas that were observed to elucidate the question of the fate of the nAChR. Figure 1 presents a schematic overview of the three distinct areas selected: the endoplasmic reticulum (ER) in red, the secretory pathway between the ER and Golgi apparatus in yellow, and the endocytic pathway after Golgi passing in purple. Subsequently, specific marker proteins were selected for each area, regarding their interaction points in the secretory pathway described in Section 1. In the best cases, these proteins are known as housekeeper proteins. The advantage of housekeeper proteins is that they are usually abundant in all cells [35,36].

The first area of our observation, the ER (red), consists of UDP-glucose:glycoprotein glucosyltransferase-1 (UGGT1) [9,37] and calnexin (CN) [38,39], which are associated with the calnexin cycle, and further synoviolin (Hrd1), which is an E3-ligase [40,41]. The binding immunoglobulin protein (BiP) [42,43,44] and Sil1 [45] are directly associated with each other [46], as well as with glyceraldehyde-3-phosphate dehydrogenase (GAPDH) in the cytosol, which is required as an indirect ATP supplier [47]. For the second area, between ER and Golgi (yellow), the following markers were selected: COPII [12] leaves the ER and consists of isoforms of Sec24A/B/C/D [14,15], and COPI is used for retrograde trafficking from the Golgi and ER–Golgi-intermediate compartments (ERGIC) [48], this latter of which consists of α/β proteins [21,22] as well as ERGIC53 [49] as a marker for ERGIC. For the third area, the endocytic pathway (purple), we selected the proteins Rab5/7 [26,50] and calmodulin [27,51], which were identified at the endocytic pathway for the secondary degradation process [52,53], while vacuolar protein sorting (vps) 26/35 [24,54] and Rab4/11 [55] were identified at the recycling pathway [52,56]. For a broader context of all chosen marker proteins, the reader is requested to read Section 1.

The concept of the cell-sonar is based on protein interactions, meaning the overexpression of the transgenic nAChR results in altered expression levels of proteins that interact with it. If no expression changes are detected, then the nAChR is not interplaying with the marker protein. In both cases, the localization of the receptor was indirectly tracked, while the nAChR itself was not directly detected. Cell-sonar was performed based on ICW, serving its advantages described in Section 1. The limitations of ICW are the co-localization of proteins, as in high-resolution microscopy applications, and a quick overview of pathway detection. The 20 selected marker proteins that are common housekeepers in Chinese hamster ovary cells—CN, BiP, Sil1, Hrd1, UGGT1, GAPDH, COPII Sec24A/B/C/D, COPIα/β, ERGIC53, Rab4/5/7/11, calmodulin, and vps26/35—were assessed regarding their expression level changes to yield a broader overview. Table 1 shows all marker proteins used, organized according to their areas of observation. Although the co-localization of proteins is not detectable, a first overview of the nAChR was possible.

### 3.2. Intracellular Tracking within the ER

**Figure 2:**
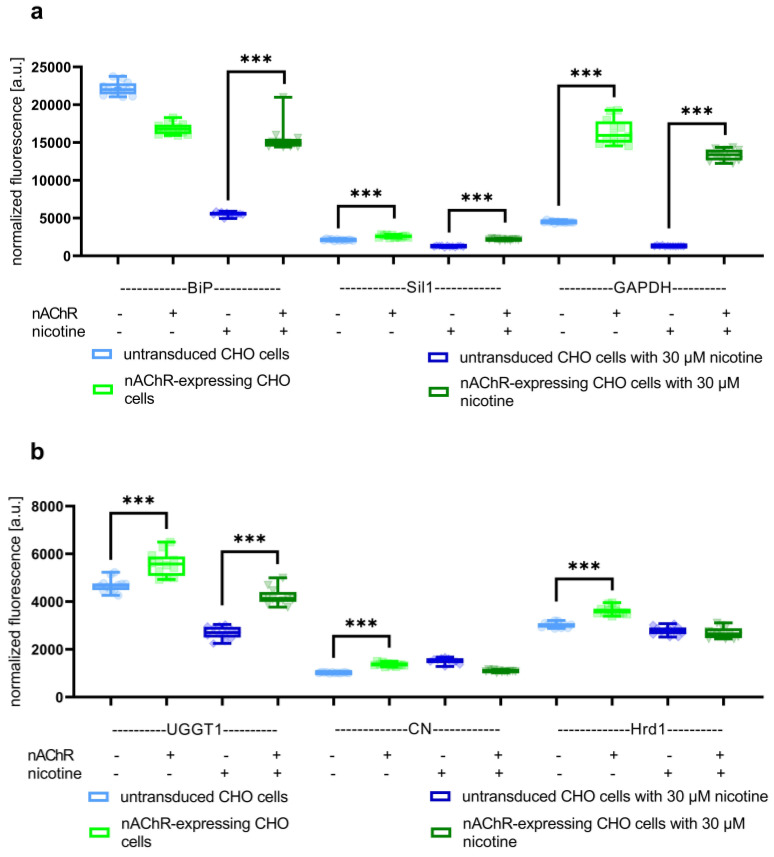
Signals from overexpression changes derived via in-cell Western using BiP, Sil1, GAPDH, UGGT1, calnexin (CN), and Hrd1 as ER markers. Boxplots of normalized fluorescence signals using (**a**) BiP, Sil1, and GAPDH as markers for ATP-dependent associations show detectable expression changes between nAChR-expressing CHO cells and untransduced controls in the presence or absence of nicotine. (**b**) The use of UGGT1, CN, and Hrd1 as markers for calnexin-cycle dependent associations also shows these different expression levels in the absence of nicotine, rather than CN and Hrd1, which were not significantly increased under nicotine treatment. The data are combined from biological triplicates, each with four technical replicates, where *** means significance with a *p*-value below 0.001.

At first, cell-sonar was used to track expression changes within the ER (red-colored area in Figure 1). Therefore, the expression levels of chaperones from the ER, UGGT1, Sil1, Hrd1, BiP, CN, and the housekeeper GAPDH were detected by ICW in untransduced and nAChR-expressing CHO cells each in the presence or absence of nicotine. This experiment should give an overview of ER homeostasis, which may also reflect the cellular stress level. All of the chaperones tested associate directly or indirectly with the nAChR, as described in Section 1. Figure 2 shows the changed expression level of the marker proteins corresponding to ATP-dependent (a) as well as calnexin cycle-dependent associations (b).

Figure 2 shows that in the absence of nicotine, all marker proteins except BiP are upregulated in nAChR-expressing cells compared to their untransduced controls. This upregulation was still seen in the presence of nicotine for Sil1, GAPDH, and UGGT1, while no significant difference was detected for CN and Hrd1. In addition, while in the absence of nicotine, BiP showed lower levels, in the presence of nicotine it showed significantly higher levels in nAChR-expressing cells compared to the untransduced controls. Overall, the ER marker proteins chosen in this study showed differences between nAChR protein overexpression as well as nicotine treatment. In conclusion, the data in Figure 2 show no increased degradation of the nAChR; in particular, under nicotine treatment, Hrd1 was not increased, suggesting no enhanced degradation process.

### 3.3. Intracellular Tracking between the ER and Golgi

**Figure 3:**
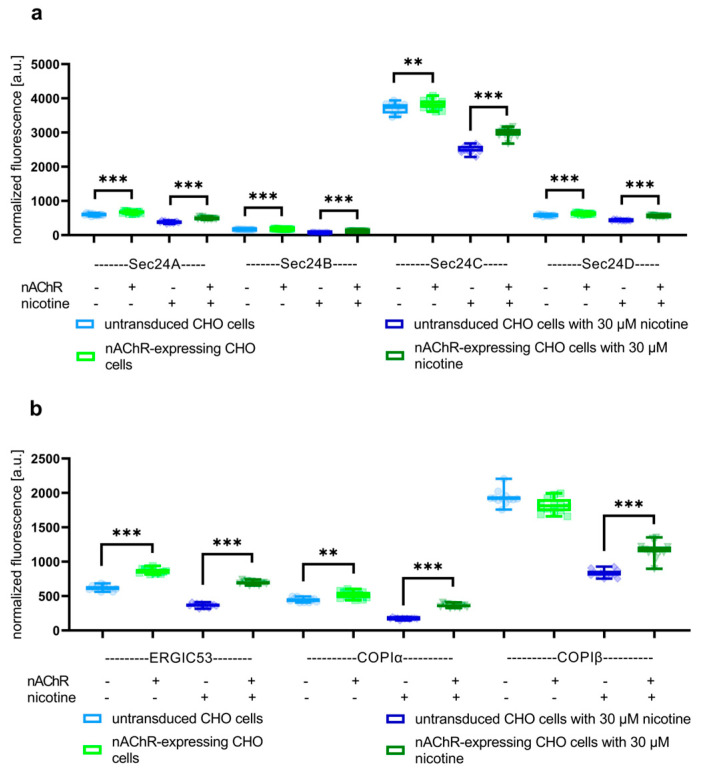
Signals derived from overexpression changes yielded by in-cell Western for Sec24A/B/C/D, using ER–Golgi-intermediate compartment (ERGIC)53 and COPIα/β as markers for transport between ER and Golgi. Boxplots of normalized fluorescence signals from studies using (**a**) Sec24A/B/C/D as markers for ER anterograde traveling in the secretory pathway show detectable expression changes between nAChR-expressing CHO cells and untransduced controls in the presence or absence of nicotine. Further, Sec24C was more significantly increased under nicotine treatment. (**b**) COPIα/β and ERGIC53, used as markers for retrograde traveling in the secretory pathway, showed more increased expression levels in the presence of nicotine, while COPIβ was not significantly increased without nicotine treatment. Data are combined from biological triplicates, each with four technical replicates; ** means significance with a *p*-value below 0.01, and *** means significance with a *p*-value below 0.001.

Secondly, cell-sonar was used to track expression changes in the secretory pathway between the ER entry site to ERGIC and Golgi (yellow-colored area in Figure 1) because the data in Figure 2 suggest that the nAChR leaves the ER at greater levels than expected. The marker proteins Sec24A/B/C/D, ERGIC53, and COPIα/β were detected by ICW in untransduced and nAChR-expressing CHO cells, each in the presence or absence of nicotine. This area should provide an overview of the possible retention that occurs in the transition from the ERGIC or Golgi back to the ER. Figure 3 shows the changed expression levels of the marker proteins corresponding to ER anterograde traveling (a) and ER retrograde traveling (b) localization in the secretory pathway.

Figure 3a,b show the levels of marker proteins for the trafficking between ER and Golgi, while (a) shows anterograde and (b) shows retrograde transport. In the absence of nicotine- and nAChR-overexpressing CHO cells, almost all markers were significantly increased compared to untransduced CHO cells. The only exception was COPIβ, which remained at about the same level. However, in the presence of nicotine treatment, all markers tested showed a significant increase im nAChR-expressing cells compared to untransduced controls. Moreover, the increase in Sec24C and COPIα/β was more pronounced in the presence than in the absence of nicotine. These data suggest that, on the one hand, nAChR enters the Golgi and, on the other hand, their retention in the ER is increased under nicotine treatment.

### 3.4. Western Blot Analysis for Confirming the Findings of Cell-Sonar That nAChR Enters Golgi

**Figure 4:**
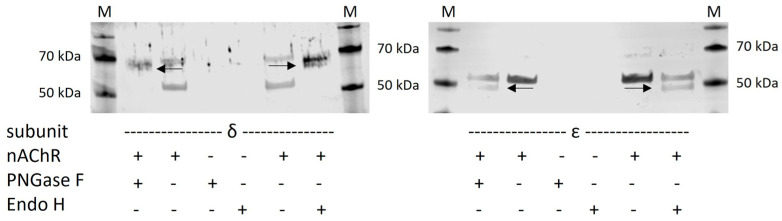
Western blot to provide evidence of the findings received by cell-sonar that nAChR enters the Golgi by glycosylation digest of the δ and ε subunit. Lysates from CHO cells expressing nAChR or of untransduced CHO control cells were treated with PNGase F and Endo H for digestion and analyzed by Western blot with subunit-specific antibodies. Black arrows indicate digested subunit bands. M indicates marker, and 50 and 70 kDa were added.

At this point, we propose that nAChR enters the Golgi apparatus, as elucidated by cell-sonar. An independent method that can be used to test Golgi entrance, which is currently the standard assay, is glycosylation digest. Thus, a glycosylation digest of the δ and ε subunits of nAChR was performed to check for Golgi entrance. Since nAChR usually matures with *N*-glycosylated glycans N76, N143, and N169 for the δ subunit, as well as N86 and N161 for the ε subunit in Golgi, this maturation to high-mannose glycans subunits was tested by digestion with glucosidase Endo H and PNGase F. After SDS PAGE and immunoblotting, the shift between the undigested subunit and the glycosylation-digested subunit is visible. Figure 4 shows the results of the glycosylation digest for the δ and ε subunits of nAChR, which is evidence of the maturation of *N*-glycans in the Golgi apparatus. Both subunits were detected by subunit-specific antibodies, and their corresponding undigested subunits and further untransduced CHO cells used as the control showed no bands.

### 3.5. Intracellular Tracking after Golgi in the Endocytic Pathway

**Figure 5:**
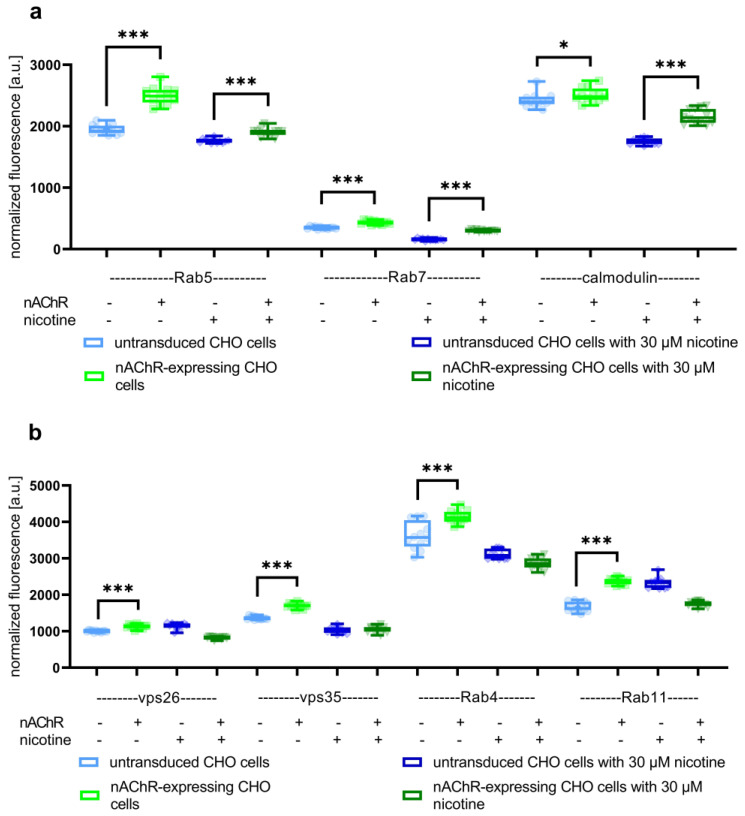
Signals from overexpression changes detected via in-cell Western analyses using Rab5, Rab7, calmodulin, vacuolar protein sorting (vps)26, vps35, Rab4, and Rab11 as markers for the endocytic pathway. Boxplots of normalized fluorescence signals using (**a**) Rab5, Rab7, and calmodulin as markers for the secondary degradation process show detectable changes in expression levels between nAChR-expressing CHO cells and untransduced controls in the absence or presence of nicotine, whereas Rab7 and calmodulin were more significantly increased under nicotine treatment. (**b**) The use of vps26, vps35, Rab4, and Rab11 as markers for the recycling/storage pathway illustrates increased expression levels in the absence of nicotine, whereas in the presence of nicotine, all storage markers were not increased. Data are combined from biological triplicates, each with four technical replicates; * means significance with a *p*-value below 0.05, and *** means significance with a *p*-value below 0.001.

Thirdly, cell-sonar was used to track expression changes in the endocytic pathway (purple-colored area in Figure 1) because, at this point of the study, we have demonstrated no increased degradation process, which explains the reduction in the cell surface of the nAChR. The expression levels of marker proteins of Rab4/5/7/11, vps26/35, and calmodulin were detected by ICW in untransduced and nAChR-expressing CHO cells, each in the presence or absence of nicotine. The endocytic area should give an overview of the destination, somewhere between the second degradation and storage. Figure 5 shows the changed expression levels of the marker proteins corresponding to the endocytic pathway to degradation (a), as well as the endocytic pathway to storage (b), localized under the cell surface.

Figure 5a,b show the levels of marker proteins for the endocytic pathway, while (a) shows the second degradation and (b) the recycling or storage pathway. In the absence of nicotine, all markers are significantly increased compared to untransduced CHO cells. In the presence of nicotine treatment, all markers for the second degradation show a significant increase for nAChR-expressing cells compared to untransduced controls. Moreover, all markers for the recycling pathway show no significant increase, and calmodulin was more expressed in the presence than in the absence of nicotine. In conclusion, the data in Figure 5 explain the lack of nAChR at the cell surface, the higher secondary degradation of the receptor under nicotine conditions, and why without nicotine the nAChR was stored in the RE.

### 3.6. FACS Analysis for Confirming the Findings of Cell-Sonar That nAChR Is in RE

**Figure 6:**
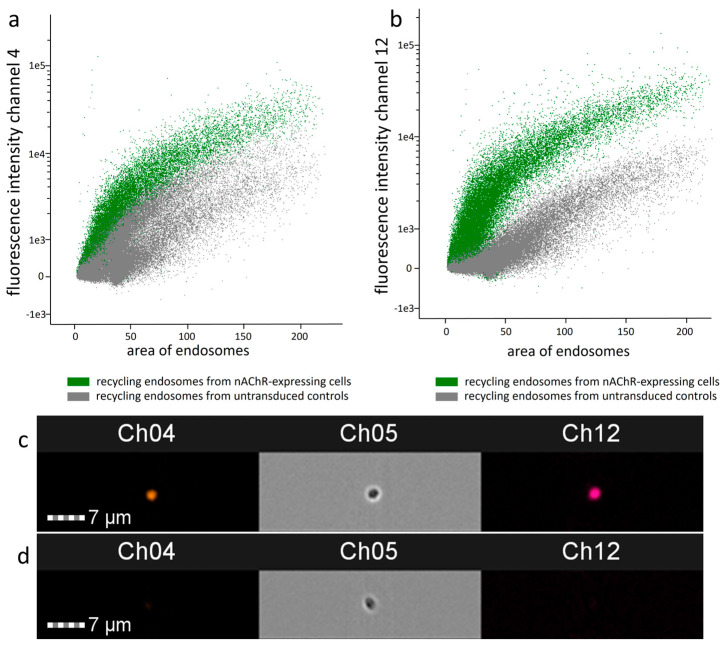
FACS analysis confirmed the findings derived by cell-sonar regarding nAChR subunits in recycling endosomes (RE) marked with Rab11 from nAChR-expressing CHO cells and untransduced control cells. (**a**) The intensity of the fluorescence signal of Channel 4 for the α1 subunit or (**b**) Channel 12 for the β1 subunit is shown by the areas of the corresponding Rab11 endosomes. (**c**) Images of Channels (Ch) 4, 5, and 12 of the same RE from nAChR-expressing CHO cells and (**d**) RE from untransduced control cells. Ch04 and Ch12 correspond to the α1 and β1 subunits, as before, and Ch05 correspond to the bright field. Data are derived from n = 3 biological experiments, each with 30,000 objects.

Based on the cell-sonar results, we propose that in the absence of nicotine, nAChR might be retained under the cell surface in RE. To provide additional independent evidence for this hypothesis, we specifically enriched RE from nAChR-expressing cells, and compared them to those of untransduced controls by flow cytometry. Therefore, we isolated Rab11-marked RE from both cell lines and performed a FACS analysis to detect the α1 and β1 subunits of nAChR within these Rab11-marked RE. The results are shown in Figure 6, while Channel 4 corresponds to the α1 and Channel 12 to the β1 subunit. As expected, RE from untransduced control cells barely showed any signals (Figure 6a,b, grey dots). In comparison, RE from nAChR-expressing cells showed fluorescence signals for both α1 and β1 subunits (Figure 6a,b, green dots). Exemplary images from one RE of nAChR-expressing cells (Figure 6c) in comparison to untransduced controls (Figure 6d) are shown.

## 4. Discussion

This study presents cell-sonar as a novel method for the intracellular tracking of a target protein by investigating protein level changes of housekeeper proteins in different subcellular compartments. Our method does not require complex or expensive equipment like high-definition microscopy. Instead, this approach uses only the detection of special marker proteins to derive an intracellular overview. The three areas of this study, (i) within ER, (ii) between ER and Golgi, and (iii) the endocytic pathway, should provide an example of how this new method can be used. Moreover, this work should demonstrate how broadly applicable the method can be, such as in the intracellular tracking of a target protein, or also for intracellular tracking following compound addition, such as nicotine. As demonstrated here, cellular tracking could be performed by comparing cell lines with and without expression of the target protein, as well as in the presence and absence of nicotine treatment. Using cell-sonar, three intracellular areas were investigated, and possible retention points for the nAChR could be identified. To validate the results gained from this new method, we compared them to already-existing, independent, and commonly accepted methods in the field, such as Western blot after glycosylation digest, and FACS analysis. These more traditional methods confirm the results derived from cell-sonar, showing its suitability. This new method provides an overview of intracellular changes at low cost, and in a short time. Thus, we think that cell-sonar may be a good starting point in the research of novel protein targets.

We propose that cell-sonar can be applied to a wide range of target proteins of interest. Moreover, every instrument or method that can detect expression level changes should be adaptable to cell-sonar. The application of their molecular chaperones, compartment marker proteins, or associated proteins is feasible, and is not limited. Moreover, the method can be reduced or expanded by use of diverse marker proteins. For example, if ER chaperoning is the main concern, only these chaperones or markers could be tested. Additionally, cellular tracking could be performed under exposure to a range of compounds.

The advantages of the new method are that it can be applied to almost any cell line and even primary cells if they are adherent. As already mentioned, this method is easy, cheap, and only requires low-cost equipment. The limitation of the approach is that the outcomes provide merely an overview. Thus, we recommend at least two marker proteins for one aspect, and more protein markers will result in a more detailed overview. Moreover, for the detection of specific pathways, detailed knowledge of the pathway itself is required. Regardless, we propose to consider the new method as a valuable extension of the current tools. We sum up the advantages and limitations of the cell-sonar method in Table 2.

**Figure 7:**
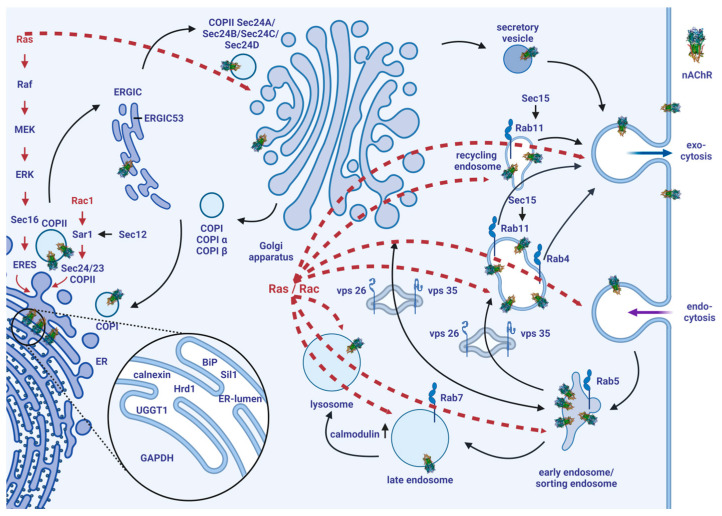
Scheme of the currently known models of intracellular traffic pathways extended by nAChR, evidenced by results from this study. See the text for a description of the model’s details. This model is based on models and findings of the following research [10,11,12,13,14,15,16,17,18,19,20,21,22,23,24,25,28,29,33,49,50,51,52,53,54,55,56,57,58,59,60,61,62]. The three chosen areas (not colored) selected as a base for the novel cell-sonar method are also shown in Figure 1. Every area is orchestrated under Ras and Rac signaling (red dashed arrows), which provides the key control of cellular protein homeostasis and is the reason for cells to dynamically react to changes, such as protein overexpression (including nAChR). Created with BioRender.com (accessed on 1 May 2024).

Our previous study [34] demonstrated that nicotine treatment increased intracellular nAChR expression only. Numerous research groups [30,31,32,33] have indicated the same phenomenon in different contexts; that cell surface presentation remains below intracellular occurrence. The main question behind this study was whether nAChR is subjected to intracellular degradation, and what other reasons could be behind these findings. The nAChR is tracked through the cell according to the steps of its biogenesis or secretory pathway. Figure 1 illustrates the cellular pathways investigated, which were extended by the results of this study to the nAChR (Figure 7).

ATP and calnexin cycle-dependent chaperones (Figure 2) were significantly increased in nAChR-expressing cells compared to untransduced controls, which might reflect a higher need for chaperones for nAChR folding in the ER. This was similar to the result in the presence of nicotine, but here, BiP instead of CN and Hrd1 is significantly overexpressed, which might be explained by the support of nicotine itself for the nAChR assembly. The marker proteins of the pathway between ER and Golgi (Figure 3a) in nAChR-expressing cells show the significantly increased anterograde transport performed by Sec24 isoforms in both the absence and presence of nicotine, suggesting that the nAChR passed the Golgi. Further, in the presence of nicotine, a more significant increase in COPIα/β was shown compared to nicotine’s absence (Figure 3b), which could imply an increase in retrograde transport under nicotine. These data, combined with the shift seen after glycosylation digest (Figure 4), show that anterograde transport is sufficient for nAChR to enter the Golgi apparatus and not be completely retained or degraded. In addition to increased markers of Hrd1 and Rab7, calmodulin (Figure 5a) was also significantly increased in nAChR-expressing cells compared to untransduced controls, suggesting that the nAChR is potentially degraded. However, the degree of secondary degradation at the lysosome seems to be higher in the presence of nicotine, as shown by a more pronounced increase in calmodulin. It is surprising that vps26/35 and Rab4/11 (Figure 5b) were significantly increased without nicotine, which suggests that nAChR might not be primarily degraded, but instead stored in RE. The results from the flow cytometric analysis of RE (Figure 6) confirm this. In contrast, under the nicotine treatment, the same protein markers were not increased as much (Figure 5b), which suggests that there might be no nAChR storage in RE, and that more are degraded in secondary degradation (Figure 5a). Our study, using the cell-sonar method, offers suggestions for the presence of nAChR in different subcellular compartments, as well as answers as to whether a specific pathway might be involved in biogenesis, correct folding, or sorting.

To ensure that the cell-sonar method works in every cell type and with every target protein, it employs universal pathways that are present in all cells. Such evolutionarily conserved signaling controls proteins that are expressed in all cells [63,64,65], such as housekeepers or chaperones. Each cell is kept in balance through proteostasis, expressing a protein stock that is healthy and functional [63]. To maintain this protein stock, there are three key aspects of the proteostasis network: biosynthesis and folding, maintenance of protein stability, and their degradation [65]. These aspects must be strictly controlled. Thus, proteins in the proteostasis network are induced by compartmental stress responses and cytosolic signaling [64,65,66]. In our study, the overexpression of nAChR is the reason for a stress response. The direct comparison to untransduced cells, which should contain the usual amounts of proteins, showed changes in molecular chaperones and other signaling pathways. The proposed cell-sonar method can be adapted to the overexpression of every other target protein, as discussed below.

The unfolded protein response (UPR) in the ER is a regulatory system that responds to intracellular changes [64], as well as overexpressed transgene target proteins. UPR is involved in the biosynthesis in the ER, and reduces ER translation. This leads to an increase in the ER functional capacity for nascent proteins [64]. Further, UPR leads to a special gene expression signal in the nucleus [67,68,69]. Stress-induced mitogen-activated protein kinase (MAPK) signaling is controlled by changes in the cells, and works outside the ER [64]. This mechanism identifies overexpressed transgene target proteins as well. Signaling, phosphorylation, and networking lead to cellular adaptation to the detected changes [64]. UPR and MAPK can work together or individually [64], and in the case of MAPK, intracellular recognition leads to their phosphorylation, which transduces signals into the nucleus for gene expression [70]. Taken together, we can infer that cells possess the capacity for the dynamic recognition and adaptation to diverse changes, such as the artificial insertion of target proteins. An evolutionarily conserved network of proteostasis allows cells to induce the molecular proteins required to manage the new conditions. CN can be cleaved by caspase 8, and the cytosolic piece of CN diffuses into the nucleus and leads to STAT3 gene expression [71]. ER-associated degradation, ER quality control, and UPR are influenced by the induction of BiP, CN, and other chaperones [72,73,74]. MAPK signaling through Ras, p38, or Rac1 transferred the stress signal into the nucleus [75,76,77]. Further scaffolds, such as p38, support MAPK cascades [78] and MAPK via p38-mediated endocytosis by recruiting Rab5 [62]. Figure 7 highlights that Ras and Rac1 signaling act together through cytoskeleton changes and cell growth [57]. This huge systemic interplay controls the recognition and reaction of target proteins in cells (red dashed arrows). It results in the fact that the overexpressed target protein, such as nAChR in our study, induces by its in-situ presence the chaperones and proteins involved in its folding and trafficking. These altered chaperones or marker protein inductions can be measured and tracked to observe the intracellular pathway of the target protein, and are the basis of the cell-sonar method.

## 5. Conclusions

To conclude, the proposed cell-sonar technique is suitable for the intracellular tracking of target proteins. New aspects of already-known mechanisms and additional pathways can be investigated. The results provide an overview of known and suggested pathways, which demonstrate the detailed and complex networking of target proteins in the cellular host. The proposed method can be adapted to various target proteins and modified according to the research questions. The additional use of added compounds allows the even more detailed elucidation of pathways.

## Figures and Tables

**Table 1:** Selected marker proteins, organized according to their areas of observation.

ER Area	Between ER and Golgi Area	Endocytic Pathway Area
Calnexin BiP Sil1 UGGT1 Hrd1 GAPDH	COPII Sec24 COPII Sec24B COPII Sec24C COPII Sec24D COPIα COPIβ ERGIC53	Rab4 Rab11 Rab5 Rab7 vps26 vps35 calmodulin

**Table 2:** Overview of advantages and limitations of the cell-sonar method.

Advantages	Limitations
Wide range of proteins adaptable	Only overview
Easy to apply, and cheap	No co-localization
Applicable to almost any cell line	At least applicable to marker proteins for one aspect
First insight into target proteins’ fate	Detailed knowledge of the pathways

## Data Availability

The datasets generated and/or analyzed during the current study are not publicly available due to military secrecy, but are available from the corresponding author on reasonable request.

## Abbreviations

a.u.	arbitrary units
BiP	binding immunoglobulin protein
CHO	Chinese hamster ovary
CN	calnexin
COP	coat protein
ER	endoplasmic reticulum
ERGIC	ER–Golgi-intermediate compartment
FACS	fluorescence-activated cell sorting
GAPDH	glyceraldehyde-3-phosphate dehydrogenase
Hrd1	synoviolin
ICW	in-cell Western
MAPK	mitogen-activated protein kinase
nAChR	nicotinic acetylcholine receptor
RE	recycling endosomes
UGGT1	UDP-glucose:glycoprotein glucosyltransferase-1
UPR	unfolded protein response
vps	vacuolar protein sorting
